# Cytochrome P450 and *O*-methyltransferase catalyze the final steps in the biosynthesis of the anti-addictive alkaloid ibogaine from *Tabernanthe iboga*

**DOI:** 10.1074/jbc.RA118.004060

**Published:** 2018-07-20

**Authors:** Scott C. Farrow, Mohamed O. Kamileen, Jessica Meades, Belinda Ameyaw, Youli Xiao, Sarah E. O'Connor

**Affiliations:** From the ‡ Department of Biological Chemistry, John Innes Centre, Norwich NR4 7UH, United Kingdom and; the § Chinese Academy of Sciences (CAS) Centre for Excellence in Molecular Plant Sciences, Shanghai Institute of Plant Physiology and Ecology, Shanghai 200000, China

**Keywords:** natural product biosynthesis, cytochrome P450, natural product biosynthesis, secondary metabolism, plant biochemistry, ibogaine, monoterpene indole alkaloid, Tabernanthe iboga, O-methyltransferase, Illumina sequencing, transcriptomics

## Abstract

Monoterpenoid indole alkaloids are a large (∼3000 members) and structurally diverse class of metabolites restricted to a limited number of plant families in the order Gentianales. *Tabernanthe iboga* or iboga (Apocynaceae) is native to western equatorial Africa and has been used in traditional medicine for centuries. Howard Lotsof is credited with bringing iboga to the attention of Western medicine through his accidental discovery that iboga can alleviate opioid withdrawal symptoms. Since this observation, iboga has been investigated for its use in the general management of addiction. We were interested in elucidating ibogaine biosynthesis to understand the unique reaction steps en route to ibogaine. Furthermore, because ibogaine is currently sourced from plant material, these studies may help improve the ibogaine supply chain through synthetic biology approaches. Here, we used next-generation sequencing to generate the first iboga transcriptome and leveraged homology-guided gene discovery to identify the penultimate hydroxylase and final *O*-methyltransferase steps in ibogaine biosynthesis, herein named ibogamine 10-hydroxylase (I10H) and noribogaine-10-*O*-methyltransferase (N10OMT). Heterologous expression in *Saccharomyces cerevisiae* (I10H) or *Escherichia coli* (N10OMT) and incubation with putative precursors, along with HPLC–MS analysis, confirmed the predicted activities of both enzymes. Moreover, high expression levels of their transcripts were detected in ibogaine-accumulating plant tissues. These discoveries coupled with our publicly available iboga transcriptome will contribute to additional gene discovery efforts and could lead to the stabilization of the global ibogaine supply chain and to the development of ibogaine as a treatment for addiction.

## Introduction

Monoterpenoid indole alkaloids (MIAs)^4^ are a large (∼3000 members) and structurally diverse class of plant-specialized metabolites restricted to a limited number of plant families in the order Gentianales with remarkable structural diversity seen in the families Rubiaceae, Loganiaceae, and Apocynaceae (1–3). *Tabernanthe iboga* or iboga (Apocynaceae) is native to western equatorial Africa and has been used in traditional medicine for centuries. Iboga rootbark is central to the Bwiti religion, including during tribal initiation ceremonies and communal masses and is said to connect members with the sacred and divine (4, 5). Howard Lotsof is credited with bringing iboga to the attention of Western medicine through his accidental discovery that iboga root extracts can alleviate opiate withdrawal symptoms, and since this observation, iboga has been investigated for its use in the treatment of broad-scale addiction (5, 6).

**Figure 1.**
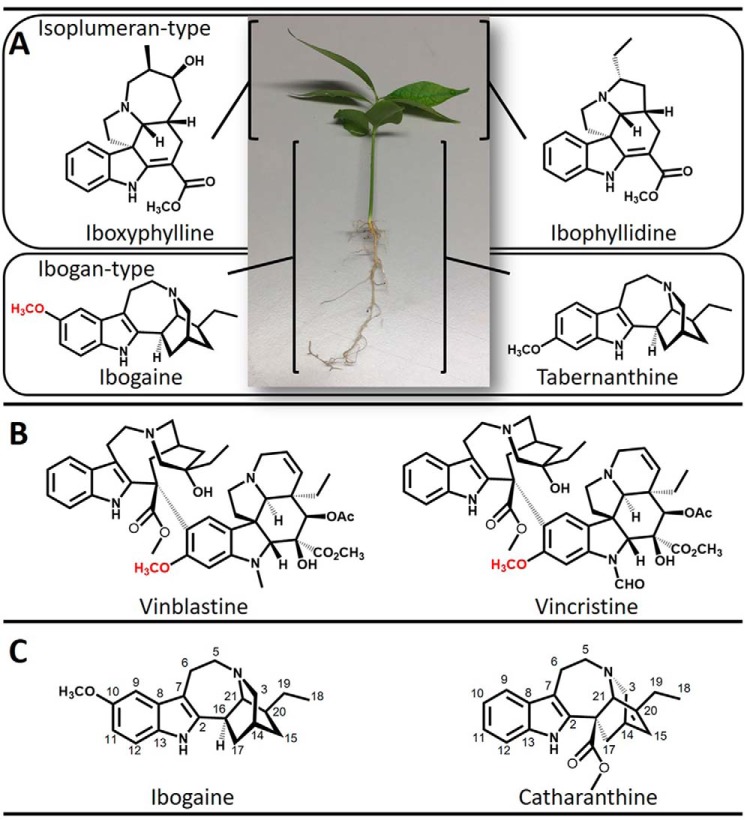
*A*, alkaloids of iboga. The principle ibogan-type alkaloids ibogaine (with C10 *O*-methyl group highlighted in *red*) and tabernanthine are abundant in the root and stems, whereas the isoplumeran-type alkaloids ibophyllidine and iboxyphylline are the core alkaloids present in leaves. *B*, anti-cancer drugs synthesized in *C. roseus* with C11 *O*-methyl group of the vindoline moiety highlighted in *red. C*, ibogaine and catharanthine are ibogan-type alkaloids belonging to opposing optical series.

*T. iboga* produces large quantities of ibogan-type MIAs, such as ibogaine and tabernanthine, in roots and stems. In contrast, the principle alkaloids of leaves are isoplumeran types, including iboxyphylline and ibophyllidine (7–10) (Fig. 1*A*). Ibogaine is the principle and active pharmacological component of iboga rootbark and shares a characteristic tryptophan/isoprenoid biosynthetic origin (11) with all other MIAs, including the anti-cancer drugs vincristine and vinblastine (1) (Fig. 1*B*). Unlike the MIA producing plants found throughout the Apocynaceae, elevated ibogaine concentrations and the preponderance of iboga-type alkaloids in general appears to be a chemotaxonomic characteristic of *T. iboga* (10, 12).

**Figure 2.**
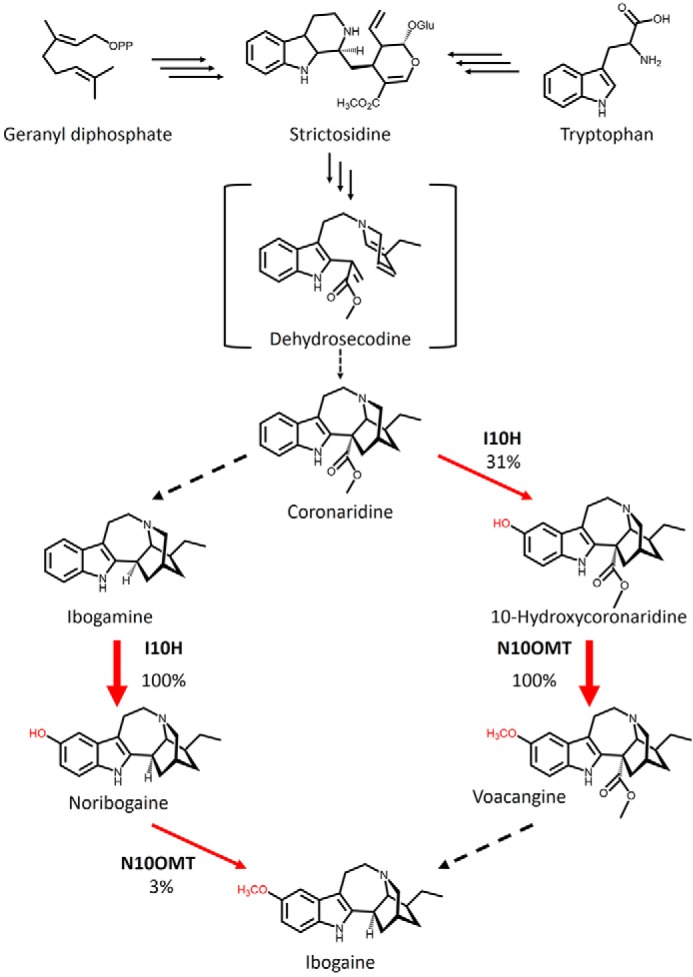
**Proposed biosynthetic pathway to ibogaine in *T. iboga*.** Tryptophan and geranyl diphosphate undergo multiple transformations (*multiple arrows*) to yield strictosidine, which undergoes multiple transformations (*multiple arrows*) to yield the hypothesized reactive intermediate dehydrosecodine. In iboga, dehydrosecodine is thought to undergo cyclization to form the (−)-ibogan scaffold and eventually (−)-coronaridine. Net decarboxylation of coronaridine yields ibogamine, which can then undergo 10- hydroxylation and 10-*O*-methylation to yield ibogaine. The pathway could also bifurcate through voacangine, whereby net decarboxylation of voacangine could lead to ibogaine. *Percentages* and *arrow thickness* indicate the relative activity of I10H and N10OMT for different substrate in the pathway, underscoring the potential routes to ibogaine. *Red arrows* represent the steps that are the focus of this study.

Interestingly, ibogaine and catharanthine, the precursor to anti-cancer drugs, are iboga-type alkaloids belonging to two distinct optical series (Fig. 1*C*). We recently solved the remaining biochemical steps en route to catharanthine in *Catharanthus roseus*, including a landmark Diels–Alder cyclization step leading to catharanthine (13). We were interested in understanding the biochemical strategies underpinning the synthesis of the disparate stereochemical ibogaine, including a potentially unique cyclization step, and the late stages of ibogaine biosynthesis that are characterized by a net decarboxylation at the 16-position, 10-hydroxylation, and 10-*O*-methylation of the predicted coronaridine precursor (Fig. 2). Considering that the commercial sources of ibogaine are primarily plant-based and that therapeutic use is contributing to overharvesting, we were keen on discovering ibogaine biosynthetic genes that could bolster current production strategies or lead to the creation of alternative production platforms through modern synthetic biology approaches. This would have the added benefit of assisting with iboga conservation efforts, a primary concern among members of the Bwiti community.

Here, we report the first transcriptome of *T. iboga*, which we used to identify a cytochrome P450 (P450), herein named ibogamine-10-hydroxylase (I10H), and an *O*-methyltransferase (OMT), herein named noribogaine-10-*O*-methyltransferase (N10OMT), that catalyze the penultimate 10-hydroxylation and final 10-*O*-methylation steps in ibogaine biosynthesis, respectively. This is the first report of enzymes involved in ibogaine biosynthesis, and the hydroxylation at the 10-position of ibogamine and coronaridine by I10H is the first example of a regiospecific hydroxylation of the iboga alkaloid scaffold. These discoveries coupled with our publicly available iboga transcriptome will contribute to additional gene discovery efforts and could lead to the stabilization of the global ibogaine supply chain and development of ibogaine as a treatment for addiction.

## Results

### Identification of P450 and OMT gene candidates

Candidates for 10-hydroxylation and 10-*O*-methylation were identified based on sequence homology to tabersonine 16-hydroxylase (T16H; P98183) and 16-hydroxytabersonine *O*-methyltransferase (16OMT; B0EXJ8) from *C. roseus* in a transcriptome of *T. iboga*. T16H and 16OMT methoxylate tabersonine to form an intermediate that is eventually incorporated into vinblastine and vincristine (Fig. 1*B*). Five P450 and six OMT candidates were identified with low to moderate (30–62%) sequence identity to T16H or 16OMT, respectively. Amino acid alignments of *T. iboga* P450s or OMTs with characterized or uncharacterized proteins show conserved canonical features of each protein type, including the absolutely conserved cysteine residue responsible for iron coordination in P450s (Fig. S1) and the multiple sequence motifs of OMTs for SAM binding (Fig. S2). Phylogenetic trees (Fig. S3) derived from these alignments showed high bootstrap support for a distinct clade of OMTs or P450s belonging to MIA-producing plants. For example, T16H is a member of the CYP71 family of P450s and formed a clade with iboga CYP71 candidates, suggesting an evolutionary linkage and possible functional similarity. Similarly, 16OMT formed a clade with OMT candidates from iboga and other MIA-producing plants, indicating a possible functional relationship.

### Identification of I10H and N10OMT

**Figure 3.**
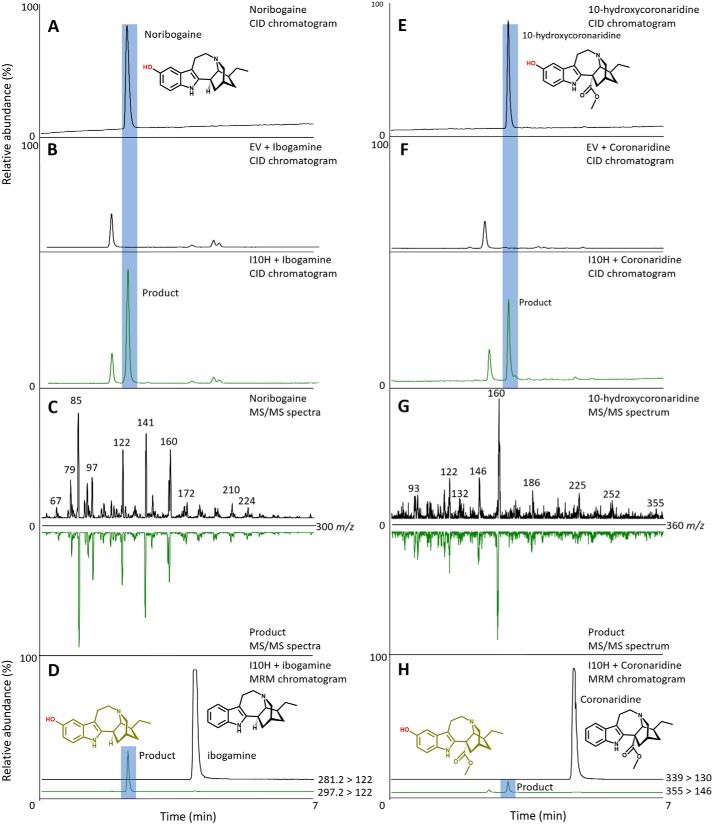
**I10H 10-hydroxylates ibogamine or coronaridine to make noribogaine or 10-hydroxycoronaridine, respectively.** Shown are HPLC–MS chromatograms and MS/MS spectra of authentic standards and products from enzyme assays with I10H and ibogamine or coronaridine. *A*, HPLC–CID chromatogram of noribogaine standard. *B*, HPLC–CID chromatogram of EV and ibogamine (*black line*) and reaction product from I10H and ibogamine assay (*green line*) that co-elutes with authentic noribogaine. *C*, MS/MS spectra of noribogaine standard (*top*) and mirrored MS/MS spectra of the product from the I10H and ibogamine assay (*bottom*). *D*, multiple-reaction monitoring of the assay with I10H and ibogamine shows the production of noribogaine under standard reaction conditions (*A*). *E*, HPLC–CID chromatogram of coronaridine standard. *F*, HPLC–CID chromatogram of EV and coronaridine (*black line*) and reaction product from I10H and coronaridine assay (*green line*) that co-elutes with 10-hydroxycoronaridine. *G*, MS/MS spectra of 10-hydroxycoronaridine (*top*) and mirrored MS/MS spectra of the product from the I10H and coronaridine assay (*bottom*). *H*, multiple-reaction monitoring of the assay with I10H and coronaridine shows the production of 10-hydroxycoronaridine under standard reaction conditions (*E*). The *unidentified peaks* in *B* and *E* are products of endogenous yeast enzymes present in empty vector and I10H microsomes.

P450s were expressed in and enriched from yeast microsomes and screened for activity with MIAs from the plumeran, isoplumeran, and ibogan subtype scaffolds (Fig. 1). HPLC–MS analysis detected a gain of 16 atomic mass units in the products of assays with P450 candidate 3 and ibogamine, coronaridine, and the synthetic substrate 18-methoxycoronaridine (18-MC). The reaction with this P450 and ibogamine yielded a product that co-eluted with and shared the same MS/MS spectrum as authentic noribogaine, indicating that the product of this reaction is noribogaine, and prompted us to name the protein I10H (Fig. 3, *A–D*). The reaction products of assays with I10H and coronaridine yielded a product that was identified as 10-hydroxycoronaridine based on a comparison of MS/MS spectra with limited published data (14) and the predicted regiospecificity of I10H for the 10-position of the ibogan scaffold (Fig. 3, *E–H*). The low concentration of product in assays with I10H and the synthetic 18-MC precluded product identification, although it is likely that hydroxylation occurs at the 10-position as with other active substrates to yield a previously unreported compound. All other P450 candidates were assayed with the same suite of substrates; however, no other reaction products were identified by HPLC–MS. Interestingly, the 11-*O*-methyl ether of ibogamine, tabernanthine, also occurs in iboga; however, none of the P450s tested showed activity toward the 11-position of ibogamine, suggesting that the enzyme responsible for this reaction might belong to a different enzyme type.

**Figure 4.**
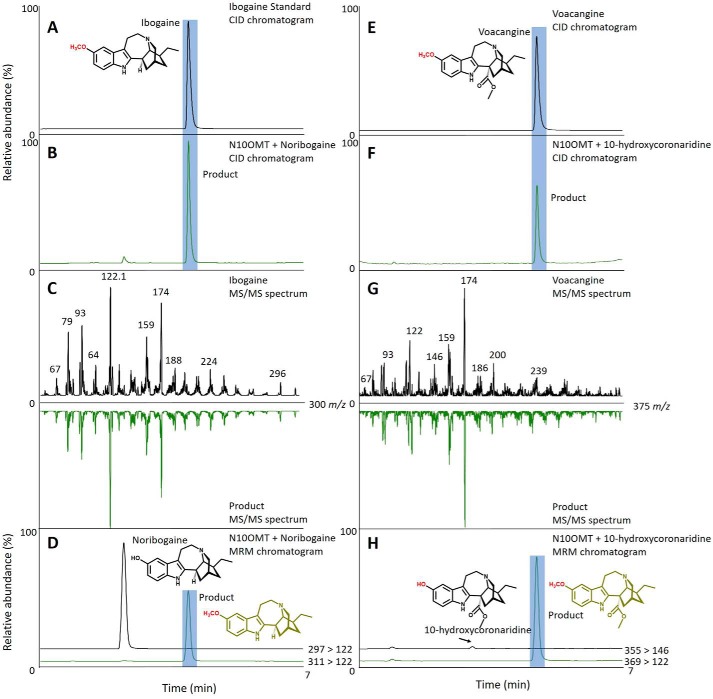
**N10OMT 10-*O*-methylates noribogaine or 10-hydroxycoronaridine to form ibogaine or voacangine, respectively.** Shown are HPLC–MS chromatograms and MS/MS spectra of standards and products from enzyme assays with N10OMT and noribogaine or 10-hydroxycoronaridine. *A*, HPLC–CID chromatogram of ibogaine standard. *B*, HPLC–CID chromatogram of reaction product from the N10OMT and noribogaine assay that co-elutes with authentic ibogaine. *C*, MS/MS spectra of ibogaine (*top*) and mirrored MS/MS spectra of the product from the N10OMT and noribogaine assay (*bottom*). *D*, multiple-reaction monitoring of the assay with N10OMT and noribogaine shows the production of ibogaine under standard reaction conditions (*A*). *E*, HPLC–CID chromatogram of 10-hydroxycoronaridine. *F*, HPLC–CID chromatogram of reaction product from N10OMT and 10-hydroxycoronaridine assay that co-elutes with voacangine. *G*, MS/MS spectra of voacangine (*top*) and mirrored MS/MS spectra of the product from the N10OMT and 10-hydroxycoronaridine assay (*bottom*). *H*, multiple-reaction monitoring of the assay with N10OMT and 10-hydroxycoronaridine (*H*) shows the production of voacangine under standard reaction conditions (*E*).

**Figure 5.**
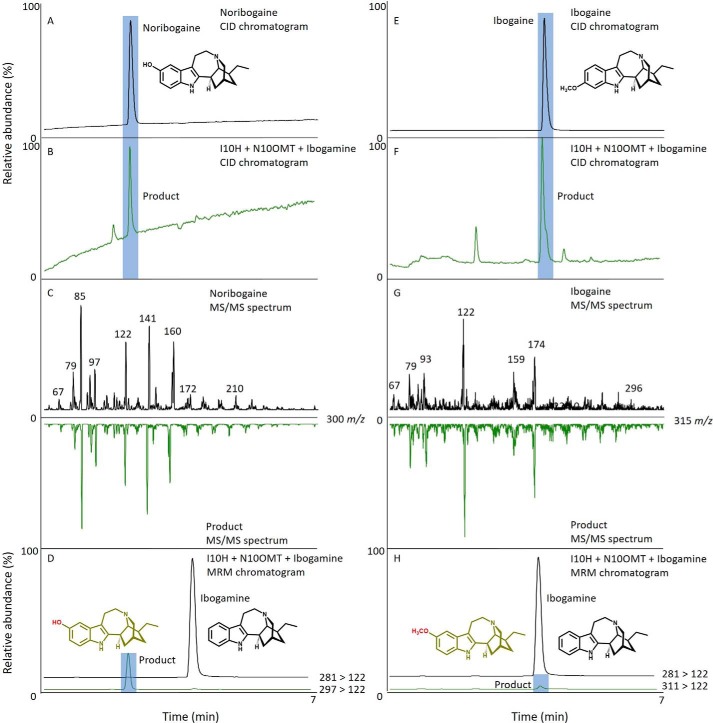
**I10H and N10OMT work in a stepwise fashion to make ibogaine.** Shown are HPLC–MS chromatograms and MS/MS spectra of standards and products from enzyme assays with I10H and N10OMT and ibogamine. *A*, HPLC–CID chromatogram of noribogaine standard. *B*, HPLC–CID chromatogram of reaction product 1 from the I10H and N10OMT and ibogamine assay that co-elutes with authentic noribogaine. *C*, MS/MS spectra of noribogaine (*top*) and mirrored MS/MS spectra of product 1 from the I10H and N10OMT and ibogamine assay (*bottom*). *D*, multiple-reaction monitoring of the assay with I10H and N10OMT and ibogamine shows the production of noribogaine under standard reaction conditions (*A*). *E*, HPLC–CID chromatogram of ibogaine. *F*, HPLC–CID chromatogram of reaction product 2 from the I10H and N10OMT and ibogamine assay that co-elutes with ibogaine. *G*, MS/MS spectra of ibogaine (*top*) and mirrored MS/MS spectra of product 2 from the I10H and N10OMT and ibogamine assay (*bottom*). *H*, multiple-reaction monitoring of the assay with I10H and N10OMT and ibogamine (*H*) shows the production of ibogaine under standard reaction conditions (*E*).

His-tagged OMTs were purified using a HisTrap Ni^2+^-affinity column and size-exclusion chromatography (Fig. S4). Purified OMTs were screened for activity with the suite of MIAs tested for P450 activity. HPLC–MS analysis detected a gain of 14 atomic mass units in the products of assays with OMT candidate 1 and both noribogaine and 10-hydroxycoronaridine. The reaction with OMT candidate 1 and noribogaine yielded a product that co-eluted with and shared the same MS/MS spectrum as authentic ibogaine, leading us to identify the product of this reaction as ibogaine, and prompted us to name the protein N10OMT (Fig. 4, *A–D*). The reaction products of assays with N10OMT and 10-hydroxycoronaridine co-eluted with and shared the same MS/MS spectrum as authentic voacangine (Fig. 4, *E–H*). Furthermore, the production of voacangine in assays with N10OMT and 10-hydroxycoronaridine supported our identification of 10-hydroxycoronaridine from assays with I10H and coronaridine (Fig. 3). To further validate our results, we co-incubated I10H and N10OMT with ibogamine. This reaction yielded two products that co-eluted with and shared the same MS/MS spectrum as authentic noribogaine or ibogaine, consistent with a stepwise biochemical reaction (Fig. 5, *A–H*). No reaction products were detected by HPLC–MS with any of the other OMT candidates and the same substrates.

### Substrate specificity

**Figure 6.**
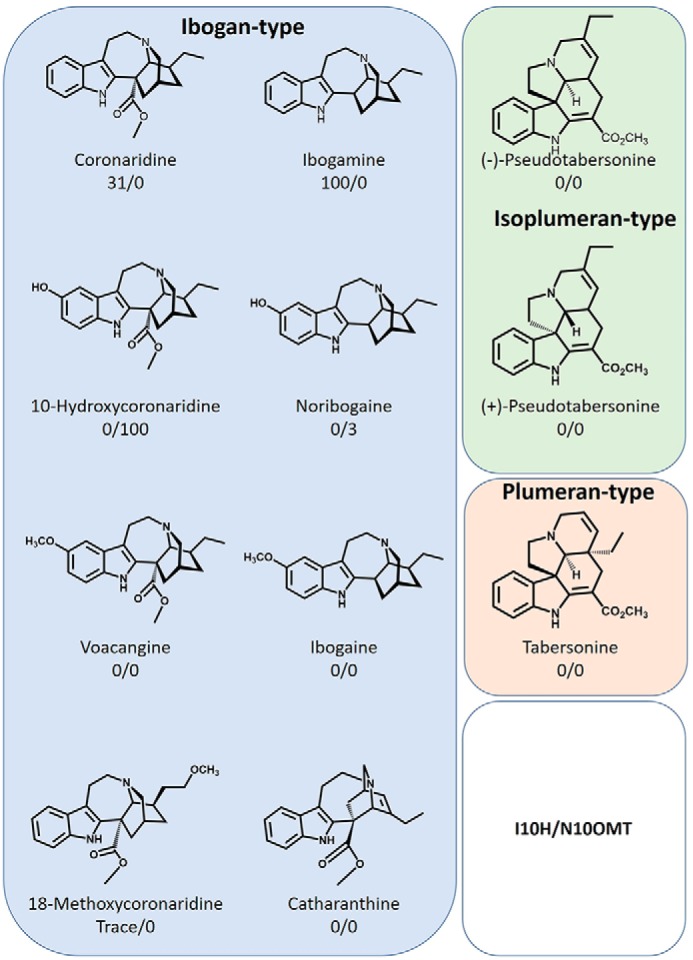
**Relative activity of I10H and N10OMT with different MIAs.** Values represent the mean percent maximum activity ± S.E. of three independent replicates for each compound compared with the substrate showing the highest turnover, which was set to 100%. Trace activity indicates less than 1% turnover.

The percent maximum conversion of I10H was highest with the ibogan-type alkaloid ibogamine (100%), but moderate (31%) activity was also detected with coronaridine. Trace hydroxylation activities below 1% were detected with the semisynthetic substrate 18-MC. N10OMT catalyzed the 10-*O*-methylation of two substrates, including relatively low turnover of noribogaine (3%) compared with high turnover of 10-hydroxycoronaridine (100%) (Fig. 6).

### Reaction kinetics and pH optimum

**Figure 7.**
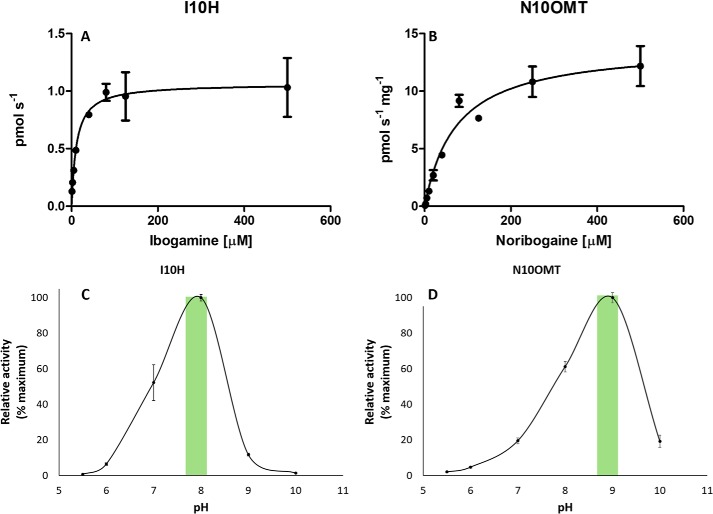
**Enzymatic activity of I10H and N10OMT.** *A* and *B*, steady-state enzyme kinetics of recombinant I10H (*A*) and N10OMT (*B*), using ibogamine and noribogaine, respectively, as substrates. Incubation time (25 min) and protein concentration (50 μg, N10OMT) were optimized before kinetic analyses. Values represent the mean product formation (pmol s^−1^ I10H or pmol s^−1^ mg^−1^ N10OMT) ± S.D. (*error bars*) of three independent measurements. Maximum velocity (*V*_max_) and the concentration of substrate that permits the enzyme to achieve half *V*_max_ (*K_m_*), catalytic rate (*k*_cat)_, and catalytic efficiency (*k*_cat_/*K_m_*) were determined based on Michaelis-Menten kinetics. Shown are pH optima of I10H (*C*) and N10OMT (*D*). I10H activity was determined for the conversion of ibogamine to noribogaine. N10OMT activity is based on the conversion of noribogaine to ibogaine. *C*, the overall pH optimum of oxidation (I10H) and methylation (N10OMT) is indicated by the *yellow highlight*. Values represent the mean ± S.D. of three independent measurements.

I10H exhibited a *K_m_* value of 11.4 ± 1.87 μm with ibogamine, whereas N10OMT displayed a *K_m_* value of 73.6 ± 28.5 μm with noribogaine. Approximate *V*_max_ values of 14 ± 1.84 pmol s^−1^ mg^−1^ for N10OMT were calculated using noribogaine as the substrate with a catalytic efficiency of 7.45 ± 3.05 m^−1^ s^−1^ (Table 1). Low quantities or inaccessibility of other substrates prevented additional kinetics experiments. Furthermore, the microsomal nature of I10H precluded the calculation of additional kinetic constants (Fig. 7, *A* and *B*). The pH optima for I10H with ibogamine and N10OMT with noribogaine were ∼8 and 9, respectively, which is consistent with other reported optima for P450s and OMTs (15–17) (Fig. 7, *C* and *D*).

**Table 1:** **Kinetic values for I10H and N10OMT with ibogamine and noribogaine, respectively**

Enzyme	*K_m_*	*V*_max_	*k*_cat_	*k*_cat_/*K_m_*
	μ*m*	*pmol s*^−^*^1^ mg*^−*1*^	*s*^−*1*^	*m*^−*1*^ *s*^−*1*^
I10H	11.4 ± 1.87			
N10OMT	73.6 ± 28.5	14 ± 1.84	5.48 × 10^−4^ ± 7.23 × 10^−5^	7.45 ± 3.05

### RT-qPCR analysis of I10H and N10OMT

**Figure 8.**
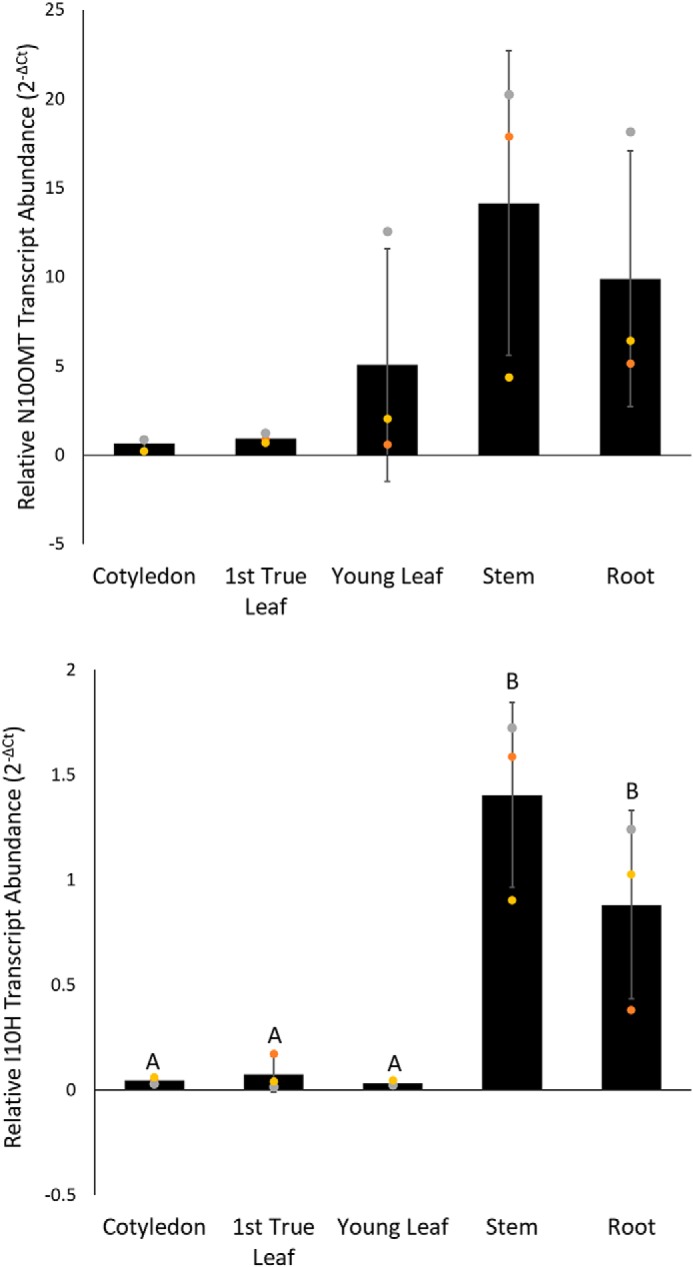
**Relative transcript abundance of I10H and N10OMT in *T. iboga* plant organs.** Real-time quantitative PCR was used to quantify the relative transcript abundance in root, stem, first true leaf, and young leaf and cotyledon of iboga seedlings. Data were calculated in Excel using 2^−Δ*Ct*^ and expressed as the mean ± S.D. (*error bars*). Each *data point* is indicated with a *gray*, *gold*, or *orange* marker. Three biological replicates and three technical replicates were analyzed for each gene using the reference gene: N2227-like family protein, *N2227*. Efficiencies for all primer sets were approximately equal and always >90%. Values with the *same letter* are not significantly different (ANOVA with Tukey PHT, *p* > 0.05).

The gene expression of I10H and N10OMT in different organs of *T. iboga* were examined using RT-qPCR (Fig. 8). I10H expression was highest in the root and stem compared with leaf and cotyledon, which showed significantly lower expression (ANOVA with Tukey PHT, *p* < 0.05). Gene expression of N10OMT was highest in stem and root followed by young leaf, first true leaf, and cotyledon; however, N10OMT was not significantly different in any of the tissues (ANOVA with Tukey PHT, *p* < 0.05).

### Metabolite analysis of iboga plant organs

**Figure 9.**
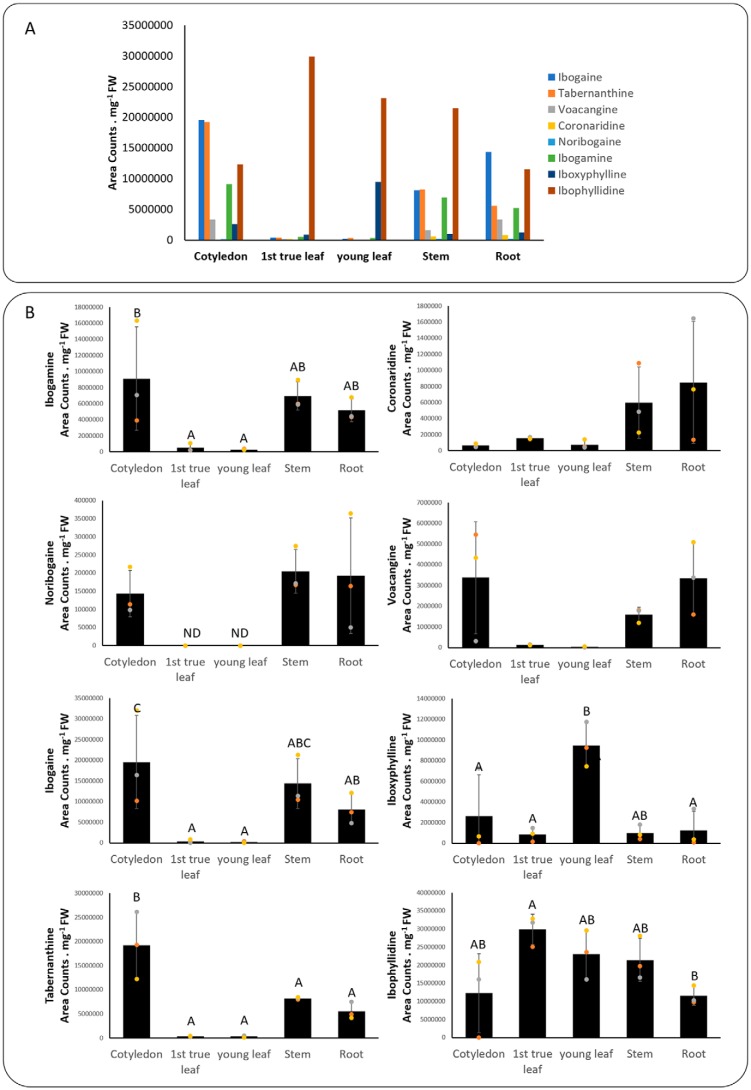
**Relative alkaloid concentration of selected MIAs from organs of *T. iboga*.** *A*, relative quantity of all reported alkaloids in each tissue. *B*, relative quantity of individual alkaloids in each tissue. Values represent the mean of three biological replicates ± S.D. (*error bars*). Values with the *same letters* are not significantly different (*p* < 0.05) using a one-way ANOVA with Tukey PHT (*n* = 3).

Selected alkaloids were profiled using HPLC–MS and displayed a tissue-specific distribution comparable with previous reports (7–10). In general, iboga-type alkaloids were highest in root, stem, and cotyledon, with the exception of coronaridine, which was low in cotyledon, and noribogaine, which was low in all of these tissues. Alternatively, these compounds were markedly lower or not detected in leaf tissue, where the distinguishing alkaloids were tentatively identified as ibophyllidine and iboxyphylline based on previous reports (7, 10) (Fig. 9).

## Discussion

*T. iboga* (iboga) is the main commercial source of ibogaine, the pharmacologically active alkaloid of iboga rootbark that is being investigated for its anti-addictive properties (6). Because ibogaine is currently sourced from plant material, we are interested in simultaneously contributing to improvements in the ibogaine supply chain and developing new synthetic biology applications for gaining access to novel small molecule therapeutics based on the ibogaine scaffold. Here, we report the first insights into ibogaine by reporting the transcriptome for *T. iboga* and the subsequent discovery of the decorating enzymes that modify the indole scaffold with a methoxy group.

We used next-generation sequencing to generate an iboga transcriptome and leveraged homology-dependent gene discovery to identify five cytochromes P450 and six OMT candidates that shared low (30%) to moderate (62%) amino acid sequence identity with tabersonine 16-hydroxylase or 16-hydroxytabersonine-*O*-methyltransferase, enzymes involved in vinblastine biosynthesis in *C. roseus*, the only other Apocynaceae plant for which an alkaloid hydroxylase/methyl transferase pair has been reported (Fig. 1*B*). We expressed these candidates in *S. cerevisiae* (P450s) or *Escherichia coli* (OMTs) and tested them with available substrates. Although the vinblastine precursor, tabersonine, that is hydroxylated does not chemically resemble ibogaine, one P450 with homology to T16H showed hydroxylase activity with ibogamine (Fig. 3, *A–D*), suggesting that it was responsible for the penultimate step in ibogaine biosynthesis. Similarly, one OMT showed *O*-methylation activity toward the product of this hydroxylase, noribogaine (Fig. 4, *A–D*), demonstrating its ability to catalyze the final step in ibogaine biosynthesis. When both proteins were incubated with ibogamine, we observed the production of two products consistent with authentic noribogaine or ibogaine, respectively (Fig. 5, *A–H*). The regiospecificity of both proteins for the 10-position of the ibogan scaffold prompted us to rename these proteins ibogamine-10-hydroxylase (I10H) and noribogaine-10-*O*- methyltransferase (N10OMT). I10H and N10OMT failed to turnover any other MIA that was tested.

Notably, this is the first example of a regiospecific 10-hydroxylation of an indole alkaloid scaffold and also the first example of a hydroxylation of the iboga alkaloid motif. I10H is therefore an important addition to the biocatalytic toolbox for alkaloid derivatization. For example, when compared with ibogaine, the 10-hydroxylated derivative, noribogaine, has shown promising results for treating addiction without some of the cardiac side effects (18).

As such, I10H could be employed biocatalytically to produce this therapeutic and analogues thereof. Similarly, the trace 10-hydroxylase activity of I10H with the semisynthetic ibogaine analogue 18-MC, which also has distinct activity profiles, could be improved by protein engineering, leading to new-to-nature ibogaine analogues with potentially novel biological activities. Sequence alignments of I10H with other alkaloid hydroxylases did not reveal any obvious amino acids responsible for switching of substrate and regioselectivity, highlighting the challenges of engineering this class of enzyme.

The roles of I10H and N10OMT in ibogaine biosynthesis *in planta* are supported by both their high expression levels in iboga tissues accumulating ibogaine (Fig. 8) and the high affinity (11 μm) of I10H and moderate affinity (73 μm) of N10OMT with ibogamine or noribogaine, respectively. However, it is important to note that the 10-hydroxylation activity of I10H and 10-*O*-methylation activity of N10OMT with the alternative substrate coronaridine and 10-hydroxycoronaridine, respectively, coupled with the presence of the downstream product voacangine in iboga, strongly suggest that I10H and N10OMT have dual roles in two branches of ibogaine biosynthesis (Fig. 2). We speculate that the decarboxylation of both coronaridine and voacangine could contribute to ibogaine concentrations in the plant. This hypothesis is underscored when comparing the relative activity of N10OMT with 10-hydroxycoronaridine and noribogaine and I10H with coronaridine and ibogamine, which, taken together, suggests that the pathway proceeds through both coronaridine and voacangine. This would be analogous to morphine biosynthesis, where thebaine bifurcates via 6-*O* or 3-*O*-demethylation to form codeinone or oripavine, respectively, both en route to morphine (19). Furthermore, considering the higher content of voacangine relative to ibogaine in members of the *Tabernaemontana* and *Voacanga* genera, it will be interesting to test the substrate preference of I10H homologs from related plants to pinpoint any chemotaxonomic differences of these P450s among species. However, although we tested six OMTs with moderate to high sequence identity to 16OMT from *Catharanthus roseus*, we cannot absolutely discount an additional OMT with higher relative activity for noribogaine compared with 10- hydroxycoronaridine.

With the rise of the global opiate drug epidemic, treatment of opiate addiction is a growing concern. To date, there are few effective treatments for opiate addiction, and this has spawned research into psychedelic compounds with the potential for treating addiction as well as mental illnesses. In this context, ibogaine has shown promising results for the treatment of opiate and broad-scale addiction; however, the stigma associated with psychedelic medicines like ibogaine is a major hurdle for its acceptance as a mainstream treatment option. Furthermore, there are reports of potentially harmful side effects of ibogaine use. In this regard, discovery of ibogaine biosynthetic genes has the potential to develop a synthetic biology application that could streamline production strategies or create access to new-to-nature compounds that treat addiction without some of the side effects. Moreover, iboga also produces isoplumeran-type MIAs, such as ibophyllidine and iboxyphylline (Fig. 1) (20), and the transcriptomic data reported here will also be key for elucidating the enzymatic underpinnings of these unique backbones. In sum, discovery of I10H and N10OMT expands the toolkit of functionalizing enzymes for the aromatic ring of MIAs, furthering the potential for tailor-made therapeutics.

## Experimental procedures

### Plant material

*T. iboga* seeds were acquired from the global ibogaine therapy alliance in May of 2014. Seeds were imbibed with warm (37 °C), sterile water for 48 h and sown in a matrix consisting of coconut coir, sharp sand, and perlite (5:4:1). Seeds were kept moist and at a constant temperature (30 °C) until radicle emergence (2–4 weeks). Seedlings were grown at a constant temperature (30 °C) under a 12-h LED light cycle (Kyson LED grow light) and watered every 2 days with 50 ml of sterile water. The growing regime was maintained until the emergence of the first leaves, whereby plants were supplemented with 50 ml of fertilizer (BIO-BIZZ) biweekly.

### Chemicals and reagents

All solvents used for extractions and preparative HPLC were of HPLC grade, whereas solvents for HPLC–MS analysis were of MS grade. All solvents were purchased from Fisher. Ibogaine, noribogaine, ibogamine, voacangine, and 18-methoxycoronaridine were gifts from Dr. Kenneth Alper (NYU Langone Health). Coronaridine was isolated from the stem bark and roots of *Tabernaemontana divaricata* with modifications to the method described previously (21). Briefly, plant material was macerated in methanol, and the extract was filtered through Whatman paper and dried *in vacuo*. The residue was resuspended in 0.01 m HCl and extracted with ethyl acetate to remove hydrophobic impurities. The aqueous extract was adjusted to pH 10 (4 m potassium carbonate), and alkaloids were extracted with ethyl acetate. The ethyl acetate fraction was dried *in vacuo* and resuspended in methanol, and coronaridine was purified using semipreparative HPLC using a Dionex Ultimate 3000 HPLC and UV detector (284 nm).

Coronaridine was eluted from a Waters XBridge semipreparative C18 column (10 × 200 mm, 5 μm in a gradient of 0.1% ammonia and acetonitrile beginning with a 5-min hold period (80:20, 0.1% ammonia/acetonitrile) and changing linearly to 50% acetonitrile in 10 min, 65% acetonitrile by 60 min, 75% acetonitrile by 62 min, and 95% acetonitrile by 62.1 min. Conditions remained at 95% acetonitrile for 2 min, and the column was re-equilibrated in starting conditions for 6 min. The fraction containing coronaridine was dried in a GeneVac (SP Scientific) and resuspended in methanol. The concentration of coronaridine was calculated using the molar extinction coefficient calculated previously (22). The identification of coronaridine was confirmed using high- resolution MS and ^1^H/^13^C NMR (Table S3). 10-Hydroxycoronaridine was prepared enzymatically by feeding coronaridine to *S. cerevisiae* expressing I10H. Briefly, a yeast starter culture was grown overnight in synthetic complete dropout medium (−leucine) with 2% glucose. I10H expression was induced by adding 100 μl of starter culture to 1 ml of synthetic complete dropout medium (−leucine) containing 0.2% glucose, 1.8% galactose, 100 mm HEPES, pH 7.5, and approximately 350 μg of coronaridine. Cultures were grown for an additional 48 h, whereby yeast was pelleted and the supernatant was collected. The pellet was extracted with 500 μl of MeOH and vigorous vortexing for 1 min, whereby debris was pelleted by centrifugation and the supernatants were pooled. The supernatant was dried under vacuum in a GeneVac, and 10-hydroxycoronaridine was purified via semipreparative chromatography according to the HPLC method above. The concentration of 10-hydroxycoronaridine was calculated using the molar extinction coefficient calculated previously (14). All gene and fragment amplifications were performed using Platinum Superfi polymerase, whereas colony PCRs were performed using Phire II master mix (Thermo Fisher Scientific). PCR product purifications were performed using the MachereyNagel PCR cleanup kit. Plasmid purifications were performed using Promega Wizard minipreps. cDNA was prepared using Superscript IV VILO master mix and ezDNase (Thermo Fisher Scientific). RT-qPCR was performed using the Sensi-FAST SYBR No-ROX kit (Bioline). The In-fusion HD cloning kit was from Clontech.

### T. iboga transcriptomics and library construction

Iboga seedlings (3 months) were ground to a fine powder under liquid nitrogen using a tissue lyser (Qiagen). RNA was extracted from 100 mg of powdered tissue using a modified CTAB method (23). RNA quality was assessed by visualizing 28S and 18S quality on an agarose gel and UV absorption ratios, where only samples with ratios above 2.0 (260/280 nm) and 2.2 (260/230 nm) were used. RNA was shipped to BGI (Hong Kong) for quality control, cDNA library construction, sequencing (HiSEQ4000 with PE150), and *de novo* transcriptome assembly.

### Gene isolation, phylogenetic analysis, and expression vector construction

*T. iboga* leaves, cotyledons, stems, and roots were flash-frozen in liquid nitrogen and powdered with the assistance of a tissue lyser. RNA was extracted from powdered tissue using the CTAB method described previously (23), and cDNA was synthesized from a mixture of root, stem, cotyledon, and leaf RNA using SuperScript IV VILO master mix with ezDNAse according to the manufacturer's protocol. Translated *T. iboga* transcriptome databases were searched using T16H (P98183) and 16OMT (B0EXJ8) protein sequences as queries to identify candidates for 10-hydroxylation of ibogamine and 10-*O*-methylation of noribogaine. Amino acid sequence alignments were performed with MAFFT (24), and phylogenetic relationships were analyzed using Geneious 9 (Biomatters Ltd., Auckland, New Zealand). Five P450 and six OMT cDNAs were amplified by RT-PCR with SuperFi DNA polymerase using primers with flanking In-fusion sites (Table S1). P450 and OMT PCR products were ligated into the pESC-leu2Δ (P450s) vector containing a cytochrome P450 reductase from *Artemesia annua* (25) or pOPINF (OMTs) using the manufacturer's In-fusion protocol. Assembled vectors were transformed into Stellar-competent cells (Clontech), whereby positive transformation was confirmed using colony PCR with the primers listed in Table S1. Sequences were confirmed using the Eurofins sequencing service (Clontech). Sequence-confirmed plasmids were transformed into *S. cerevisiae* strain PEP4 (P450s) or *E. coli* expression strain SoluBL21 (OMTs).

### Protein expression and purification

Starter cultures of *E. coli* harboring OMT:pOPINF constructs were grown overnight at 37 °C. A 1:100 dilution was made by adding overnight culture to 1 liter of 2YT medium containing 50 μg ml^−1^ carbenicillin. Protein expression was induced at *A*_600_ = 0.7 by adding isopropyl 1-thio-β-d-galactopyranoside to a final concentration of 300 μm. Protein expression continued at 18 °C for 16 h, whereby cells were harvested by centrifugation (4000 rpm). Bacterial pellets were resuspended in 50 ml of protein extraction buffer (A1 buffer, 50 mm Tris, 50 mm glycine, 5% glycerol (v/v), 0.5 m NaCl, 20 mm imidazole) containing 10 mg of lysozyme (Sigma, L6876) and one tablet of cOmplete EDTA-free protease inhibitor (Roche Applied Science). Cells were lysed at 4 °C using a cell disruptor (25 KPSI) and centrifuged at 35,000 × *g* to remove insoluble debris. The supernatant was filtered through a 0.45-μm glass syringe filter and applied to a HisTrap nickel-affinity column with the assistance of an AKTA pure FPLC.

The protein-charged resin was washed with 20 column volumes of A1 buffer, and protein was eluted with B1 buffer (A1 buffer + imidazole to a final concentration of 500 mm). OMTs were subjected to a further purification step on an SEC-1660 gel filtration column and buffer-exchanged in A4 buffer (20 mm HEPES, 150 mm NaCl, pH 7.5). Each OMT (2 μg) was separated by SDS-PAGE to assess protein yield and purity (Fig. S4). Final protein concentrations used for enzyme assays were calculated using the theoretical protein extinction coefficient, molecular weight, and absorbance on a nanodrop.

Freshly transformed yeast cultures harboring pESC-leu2Δ constructs were grown on an orbital shaker (250 rpm) to log phase in 50 ml of synthetic complete dropout medium lacking leucine (SC−leu) and 2% (w/v) glucose. Cultures were subsequently centrifuged (4000 × *g*) and resuspended in 1 liter of protein induction medium containing SC−leu supplemented with 0.2% (w/v) glucose and 1.8% (w/v) galactose. Cultures were grown for an additional 24 h at 30 °C, and cells were harvested by centrifugation. Microsomes were prepared based on a standard protocol. Briefly, after initial treatment with TEK buffer (5 mm Tris-HCl, pH 7.5, 0.5 mm EDTA, 100 mm NaCl, 20 mm KCl, 10 mm MgCl*_2_*), cells were resuspended in 5 ml of cold TESB (50 mm Tris-HCl, pH 7.5, 150 mm NaCl, sorbitol) in a 50-ml tube. Yeast cells were lysed at 4 °C with a cell disruptor (25 KPSI), and insoluble debris was removed by centrifugation at 30,000 × *g*. The supernatant was subjected to sucrose gradient separation of microsomes by ultracentrifugation for 1 h at 35,000 × *g* and 4 °C. Microsomal pellets were resuspended in 1 ml of TEG buffer (pH 7.5) and assayed immediately or stored at −80 for future use. I10H expression was confirmed using proteomics of microsomal preparations, whereby two unique peptides were detected (Peptide 1: NVFQETGNVDETK; mass, 1479.6842 Da; Peptide 2: NVFQETGNVDETKLGELK; mass, 2020.0113 Da).

### N10OMT and I10H assays

A selection of available MIAs were screened in triplicate as potential substrates for OMTs and P450s using a standardized assay. For each screen, 50 μg of purified and desalted OMT or 20 μl of purified microsome containing the P450 was incubated for 16 h at 30 °C with 25 μm candidate substrate and 100 μm SAM (OMT) or 100 μm NADPH (P450), in 50 mm HEPES, pH 7, in a total reaction volume of 100 μl. Denatured proteins were used as negative controls. Assays were quenched with 2 volumes of methanol, and samples were reduced to dryness in a GeneVac and resuspended in 1.5 ml of HPLC running buffer (0.1% formic acid/methanol, 50:50 (v/v)). MIAs accepted as substrates were used to determine relative conversion rates using identical assay conditions except for an incubation time of 25 min, which was within the linear range of each assay, a final alkaloid substrate concentration of 20 μm, and a total reaction volume of 100 μl. Assay pH was also adjusted to 8 for P450 assays and 9 for OMT assays based on empirical pH optimum. Samples were diluted to 1.5 ml with HPLC running buffer and analyzed by HPLC–MS. Noribogaine and ibogamine were used to determine kinetic parameters for N10OMT and I10H, respectively, at concentrations up to 500 μm. Samples were diluted with HPLC running buffer to a final substrate concentration of 1 μm for analysis by HPLC–MS.

### Analysis of enzyme assays

Enzyme assays were analyzed by HPLC–MS using an Acquity UPLC coupled with a XEVO TQS (Waters). For substrate range and relative activity analyses, 1 μl of sample was injected onto an Acquity BEH C18 column (1.7 μm, 2.1 × 50 mm), and analytes were eluted with a gradient of running buffer (0.1% formic acid; solvent A) and acetonitrile (solvent B). The flow rate was 600 μl min^−1^, and the gradient began with 2% solvent B and increased linearly to 98% solvent B by 7 min. The mobile phase composition remained constant until 8.1 min, at which time it returned immediately to 0% solvent B followed by a 2-min re-equilibration period. Analytes were subjected to positive electrospray ionization using source conditions optimized for MIAs (capillary voltage, 3.0 kV; source temperature, 150 °C; desolvation temperature, 500 °C; cone gas, 50 liters h^−1^; desolvation gas, 800 liters h^−1^) and were subsequently detected by multiple-reaction monitoring (MRM) with conditions for each analyte (Table 2). MRM conditions were determined using the Waters Intellistart software by injecting standards into the Waters mass spectrometer. Transitions giving the highest intensity peaks were selected for MRM panels in Figs. 3–5. Relative enzyme activity was calculated as the percentage turnover of each substrate using the formula, product peak area/(substrate peak area + product peak area) × 100. Subsequently, the compound with the highest turnover for each protein was set to 100%, and the detected activity with all other substrates was expressed as a percentage of the maximum. For kinetic parameter analyses, a five-point calibration curve of noribogaine or ibogaine (500 pm to 5 μm) was established to semiquantitatively determine the amount of product formed per unit of time. Kinetic constants were calculated based on Michaelis–Menten kinetics using Prism version 5 (GraphPad Software Inc., La Jolla, CA).

**Table 2:** **Parameters used for multiple-reaction monitoring**

Compound	Precursor ion *m/z*	Product ion 1 *m/z*	Product ion 2 *m/z*	Cone voltage	Collision energy
				*V*	*eV*
Voacangine	369.2	122.1	174.1	26	40
18-Hydroxycoronaridine	369.2	130.1	144.2	26	40
Coronaridine	339.2	130.1	144.2	26	30
10-Hydroxycoronaridine	355.2	146.1	160.1	26	40
Ibogamine	281.2	122.1	144.1	26	36
Noribogaine	297.2	122.1	160.1	26	30
Ibogaine	311.2	122.1	173.9	26	30

### Reaction product identification

Comparison of collision-induced dissociation (CID) spectra with empirical spectra either from authentic standards or available in the literature was used to identify each enzymatic reaction product (Figs. 3–5). CID was performed at 40 eV for each compound, and product ions were detected between 50 *m*/*z* and 5–10 atomic mass units above the *m*/*z* of the quasimolecular ion. CID was carried out using the HPLC–MS method above with the exception of changing the scan mode to MS2 scan.

### RT-qPCR

*T. iboga* root, stem, cotyledon, first true leaf, and young leaf were flash-frozen in liquid nitrogen and powdered with the assistance of a tissue lyser. RNA was extracted from each tissue using the CTAB method described previously (23). 700 ng of total RNA from each sample was treated with ezDNAse and reverse-transcribed using VILO superscript IV according to the manufacturer's protocol. Relative transcript abundance for each tissue was determined by RT-qPCR and expressed as 2^−Δ*Ct*^ on a Bio-Rad CFX96 Q-PCR instrument using the cDNA synthesized above and the primers listed in Table S2. Three biological replicates and three technical replicates were analyzed for each gene using the reference gene: N2227-like family protein, *N2227*. Efficiencies for all primer sets were approximately equal and always >90%. Analysis was performed in Excel.

### Metabolite analysis

A frozen and pulverized aliquot of each tissue was collected in preweighed microcentrifuge tubes and extracted with 1 ml of methanol added to 1 μg ml^−1^ ajmaline. Alkaloids were extracted for 1 min with vigorous vortexing and sonication followed by centrifugation (14,000 × *g* for 1 min) to remove insoluble debris. The supernatant was passed through a 0.22-μm membrane and diluted 1:4 with 0.1% formic acid/methanol (50:50, v/v) in a 2-ml glass autosampler vial. HPLC–MS analysis was performed on a Shimadzu LCMS-IT-TOF mass spectrometer coupled to a Nexera 2 chromatographic system. Chromatographic separation was carried out using a Kinetex XB-C18 column (2.6 μm, 100 × 2.10 mm; Phenomenex) using a binary solvent system consisting of solvent A (0.1% formic acid) and solvent B (acetonitrile). Compounds were eluted with a linear gradient of 10–30% solvent B in 5 min. The column was washed with 100% B for 1.5 min and re-equilibrated to 10% B for 2.5 min before the next injection. The flow rate was set at 600 μl min^−1^, and the injection volume was 1 μl. Mass spectrometry was performed in positive ion mode scanning from 150 to 1200 *m*/*z*. The source settings were as follows: heat block temperature, 300 °C; nebulizing gas flow, 1.4 liters min^−1^; CDL temperature, 250 °C; detector voltage, 1.6 kV. Data analysis was performed using the Shimadzu Profiling Solution software, and normalization of data was accomplished using tissue weight. Furthermore, ajmaline detection via MS did not vary beyond 5%.

## Author contributions

S. C. F. characterized all proteins, purified substrates, and coordinated all experiments. M. K. performed SDS-PAGE and protein purification. J. M. helped with initial cloning and characterization of I10H and N10OMT. B. A. helped with cloning. S. C. F. and S. E. O. wrote the manuscript. S. F., M. O. K., S. E. O., and Y. X. revised the manuscript.

## Supplementary Material

Supporting Information

## Footnotes

4 The abbreviations used are:

MIA: monoterpene indole alkaloid
18-MC: 18-methoxycoronaridine
OMT: *O*-methyltransferase
16OMT: 16-hydroxytabersonine *O*-methyltransferase
P450: cytochrome P450
I10H: ibogamine-10-hydroxylase
MRM: multiple-reaction monitoring
N10OMT: noribogaine-10-*O*-methyltransferase
T16H: tabersonine 16-hydroxylase
RT-qPCR: real-time quantitative PCR
ANOVA: analysis of variance
PHT: post hoc test
CID: collision-induced dissociation
CTAB: cetyltrimethylammonium bromide
KPSI: kilopounds per square inch.
