# Supplementary material for: Cytochrome P450 and O-methyltransferase catalyze the final steps in the biosynthesis of the anti-addictive alkaloid ibogaine from Tabernanthe iboga
Source: J Biol Chem. 2018 Jul 20;293(36):13821–33. doi: 10.1074/jbc.RA118.004060 (PMC6130943; doi:10.1074/jbc.RA118.004060)
Supplement: Supporting Information [file supp_293_36_13821__index.html]

Cytochrome P450 and O-methyltransferase catalyse the final steps in the biosynthesis of the anti-addictive alkaloid ibogaine from Tabernanthe iboga — Discovery of the final steps in ibogaine biosynthesis — Cytochrome P450 and O-methyltransferase catalyze the final steps in the biosynthesis of the anti-addictive alkaloid ibogaine from Tabernanthe iboga — Discovery of the final steps in ibogaine biosynthesis — Supporting Information 

# Cytochrome P450 and *O*-methyltransferase catalyze the final steps in the biosynthesis of the anti-addictive alkaloid ibogaine from *Tabernanthe iboga*

## Supporting Information

- Supporting Information - Supporting Figures and Tables
