# Supplementary material for: Cytochrome P450 and O-methyltransferase catalyze the final steps in the biosynthesis of the anti-addictive alkaloid ibogaine from Tabernanthe iboga
Source: J Biol Chem. 2018 Jul 20;293(36):13821–33. doi: 10.1074/jbc.RA118.004060 (PMC6130943; doi:10.1074/jbc.RA118.004060)
Supplement: Supporting Information [file supp_RA118.004060_138283_2_supp_169222_pbyng1.pdf]

Supporting Information

**Cytochrome P450 and *O*-methyltransferase catalyze the final steps in the biosynthesis of the anti-addictive alkaloid ibogaine from *Tabernanthe iboga***

Scott C. Farrow, Mohamed O. Kamileen, Jessica Meades, Belinda Ameyaw, Youli Xiao, Sarah E. O'Connor

Table of Contents:

Table S1. Primers used for gene amplification and colony PCR.

Table S2. Primers used for RT-qPCR.

Table S3. NMR spectra of Coronaridine.

Figure S1. Amino acid sequence alignment of I10H with characterized and uncharacterized P450s.

Figure S2. Amino acid sequence alignment of N10OMT with characterized and uncharacterized OMTs.

Figure S3. Phylogenetic trees of I10H and N10OMT.

Figure S4. SDS-PAGE of recombinant OMTs produced in *Escherichia coli*.

**Table S1:** Primers used for Full-length Gene amplification and colony PCR

| Gene | Strand | In-Fusion Site | Primer Sequence (5'-3') |
|---|---|---|---|
| I10H | Forward | ACCCTCACTAAAGGGCGGCCGCAACC | ATGGAGCTGATCGTCTCCTTC |
| I10H | Reverse | GTCATCCTTGTAATCCATCGATAC | TGCTTCATCCATCATTACC |
| P450-2 | Forward | ACCCTCACTAAAGGGCGGCCGCAACC | ATGGAGTTAGTCACTGCC |
| P450-2 | Reverse | GTCATCCTTGTAATCCATCGATAC | CACAGAAGAATGACTATAAGGAATTG |
| P450-3 | Forward | ACCCTCACTAAAGGGCGGCCGCAACC | GAGCTCATCTTCTTTGTCTG |
| P450-3 | Reverse | GTCATCCTTGTAATCCATCGATAC | TTGTAGATAAGAACGGGAGTG |
| P450-4 | Forward | ACCCTCACTAAAGGGCGGCCGCAACC | ATGGAGCTCATCTTTCTCTTCTG |
| P450-4 | Reverse | GTCATCCTTGTAATCCATCGATAC | GATCGCTTTTTTCAAAGAAGAAC |
| P450-5 | Forward | ACCCTCACTAAAGGGCGGCCGCAACC | ATGGAGTTCATTCTTTTCCTTTGCATTC |
| P450-5 | Reverse | GTCATCCTTGTAATCCATCGATAC | CTCCAGTTTTAGGAAAGAACC |
| N10OMT | Forward | AAGTTCTGTTTCAGGGC | CCGGACGCGAAATCTGCCGAAC |
| N10OMT | Reverse | ATGGTCTAGAAAGCTTTA | AGGATACACTTCAATGAGACTCC |
| OMT-2 | Forward | AAGTTCTGTTTCAGGGC | CCGGCAATGGTTGAGAAATCTGC |
| OMT-2 | Reverse | ATGGTCTAGAAAGCTTTA | AGGATAGACCTCAATGAGAC |
| OMT-3 | Forward | AAGTTCTGTTTCAGGGC | CCGGGAGAAGCCCAGGCTCAG |
| OMT-3 | Reverse | ATGGTCTAGAAAGCTTTA | AGGGTAGGCTTCAATGACAG |
| OMT-4 | Forward | AAGTTCTGTTTCAGGGC | CCGAGTGTAGCTTTGAATGGTG |
| OMT-4 | Reverse | ATGGTCTAGAAAGCTTTA | ATAATAAACCTCAATAAGAGATCTC |
| OMT-5 | Forward | AAGTTCTGTTTCAGGGC | CCGGCAGGAGAAGAGGAAGCTTG |
| OMT-5 | Reverse | ATGGTCTAGAAAGCTTTA | TTTAAGCAATTCCATAATCCAACTGTTG |
| OMT-6 | Forward | AAGTTCTGTTTCAGGGC | CCGGATTCTTCCCCCACAATCC |
| OMT-6 | Reverse | ATGGTCTAGAAAGCTTTA | CTTGTAGAACTCCATGATCCAC |
| | | Primers used for Colony PCR | |
| Name | Strand | Vector | Primer Sequence (5'-3') |
| GAL10-F | Forward | pESC-leu2Δ | GGTGGTAATGCCATGTAATATG |
| GAL10-R | Reverse | pESC-leu2Δ | GGCAAGGTAGACAAGCCGACAAC |
| T7-F | Forward | pOPINF | TAATACGACTCACTATAGGG |
| pOPINF-R | Reverse | pOPINF | TAGCCAGAAGTCAGATGCT |

**Table S2:** Primers used for RT-qPCR

| Gene | Strand | Primer Sequence (5'-3') |
|---|---|---|
| I10H | Forward | AGGCCTCCCTCACTGTGTCCT |
| I10H | Reverse | TGGATGGTCTGTCGGCAAAGA |
| N10OMT | Forward | AAGTGCGCTTACGATGCT |
| N10OMT | Reverse | TCTTCATTCTGGAACCACTCAC |
| N2227 | Forward | GTGAACGTGACCAGTGCTATAA |
| N2227 | Reverse | CAAGCAGGTGGACTCTCTTTAC |

**Table S3:** NMR spectra of Coronaridine

| Number | This Study $^1H$ ($J$/H) | $^{13}C$ | Reference (22) $^1H$ ($J$/Hz) | $^{13}C$ |
|---|---|---|---|---|
| 2 | - | 138.5 | - | 136.6 |
| 3 | 2.79 (2H, ddd, 8.5, 1.7, 1.7), 2.94 (2H, ddd, 2.5, 3.7, 8.5) | 54.3 | 2.81 (1H, brd), 2.89-2.92 (1H,m) | 51.6 |
| 5 | 3.40 (1H, m), 3.00-3.16 (1H, m) | 54.7 | 3.36-3.42 (1H, m), 3.15-3.23 (1H, m) | 53.1 |
| 6 | 3.00-3.16 (2H, m) | 22.8 | 2.98-3.04 (1H, m), 3.15-3.23 (1H, m) | 22.1 |
| 7 | - | 110.7 | - | 110.3 |
| 8 | - | 137.6 | - | 128.8 |
| 9 | 7.40 (1H, ddd, 7.8, 1.0, 1.0) | 118.7 | 7.47 (1H, brd) | 118.4 |
| 10 | 7.02 (1H, ddd, 7.6, 7.6, 1.2) | 122.2 | 7.08 (1H, ddd) | 119.2 |
| 11 | 6.96 (1H, ddd, 7.5, 7.5, 1.1) | 119.5 | 7.14 (1H, ddd) | 121.9 |
| 12 | 7.24 (1H, ddd, 7.9,1.0, 1.0) | 111.5 | 7.24 (1H, dd) | 110.3 |
| 13 | - | 129.6 | - | 135.5 |
| 14 | 1.85 (1H, m) | 29.0 | 1.88 (brs) | 27.4 |
| 15 | 1.12 (1H, m), 1.76 (1H, m) | 33.2 | 1.13 (1H, ddt), 1.73 (1H, m) | 32.0 |
| 16 | - | 56.4 | - | 55.1 |
| 17 | 1.92 (1H, m), 2.72 (1H, ddd, 13.1, 2.1, 2.1) | 37.0 | 1.9 (1H, ddd), 2.58 (1H, dd) | 36.5 |
| 18 | 0.91 (3H, t, 7.4) | 12.1 | 0.9 (3H,t) | 11.6 |
| 19 | 1.34-1.49 (1H, m), 1.50-1.62 (1H, m) | 28.0 | 1.4-1.47 (1H, m), 1.53-1.60 (1H, m) | 26.7 |
| 20 | 1.34-1.49 (1H, m) | 40.1 | 1.32-1.35 (1H, m) | 39.1 |
| 21 | 3.62 (1H, brs) | 57.9 | 3.56 (1H, brs) | 57.5 |
| 22 | - | 176.3 | - | 175.7 |
| $CO_2CH_3$ | 3.69 (3H, s) | 52.9 | 3.71 (1H, s) | 52.5 |

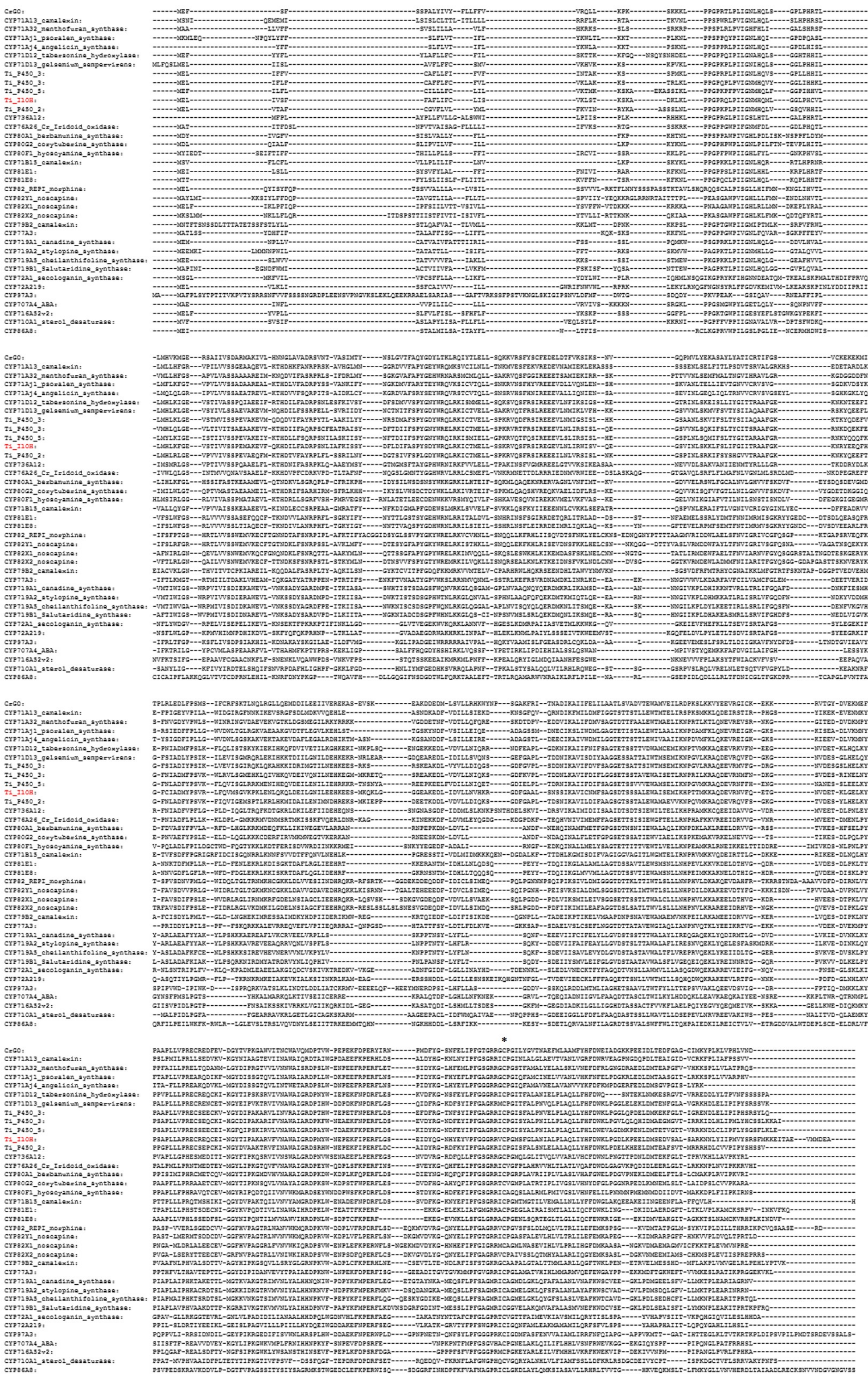

**Figure S1:** Amino acid sequence alignment of I10H with characterized and uncharacterized P450s. Asterisk indicate absolutely conserved cysteine residue for coordination of iron. I10H is highlighted in red.

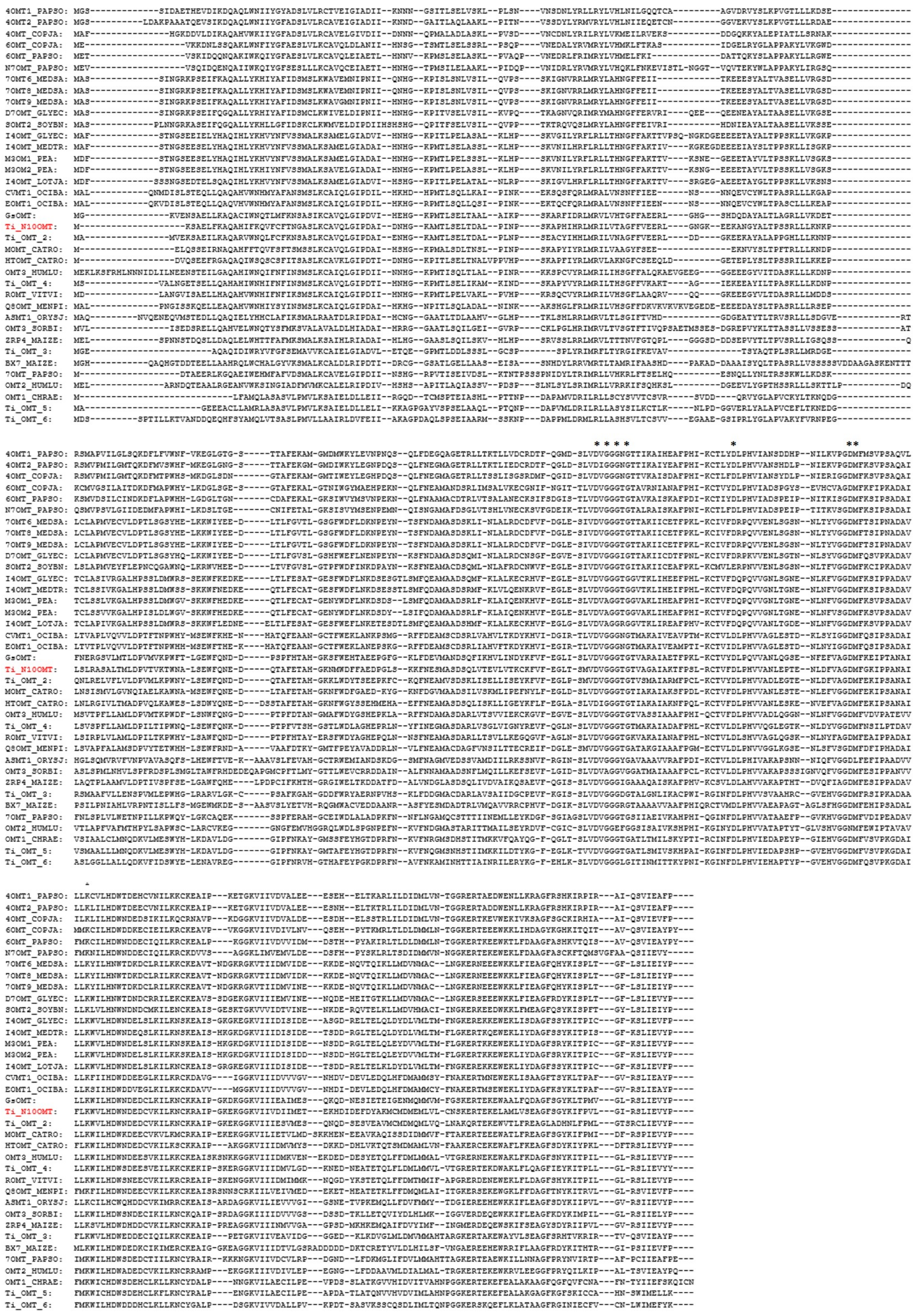

**Figure S2:** Amino acid sequence alignment of N10OMT with characterized and uncharacterized OMTs. asterisks indicate conserved residues for SAM binding. N10OMT is highlighted in red.

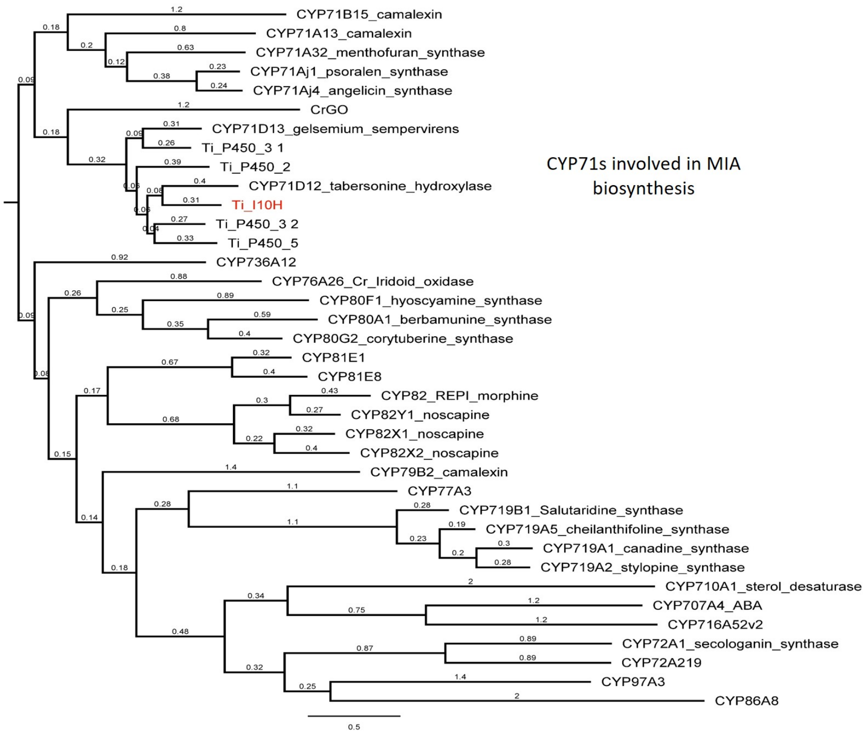


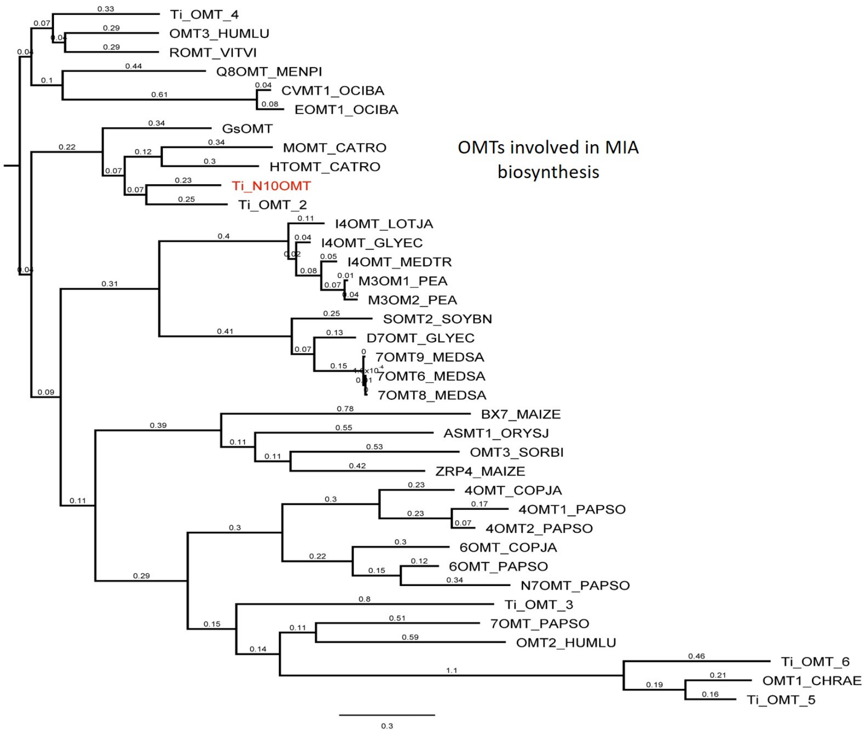


**Figure S3:** Phylogenetic trees of I10H (TOP) and N10OMT(Bottom) with characterized and uncharacterized proteins. CYP71D12 *C.roseus* tabersonine hydroxylase, T16H; HTOMT CATRO, *C.roseus* 16OMT. I10H and N10OMT are highlighted in red.

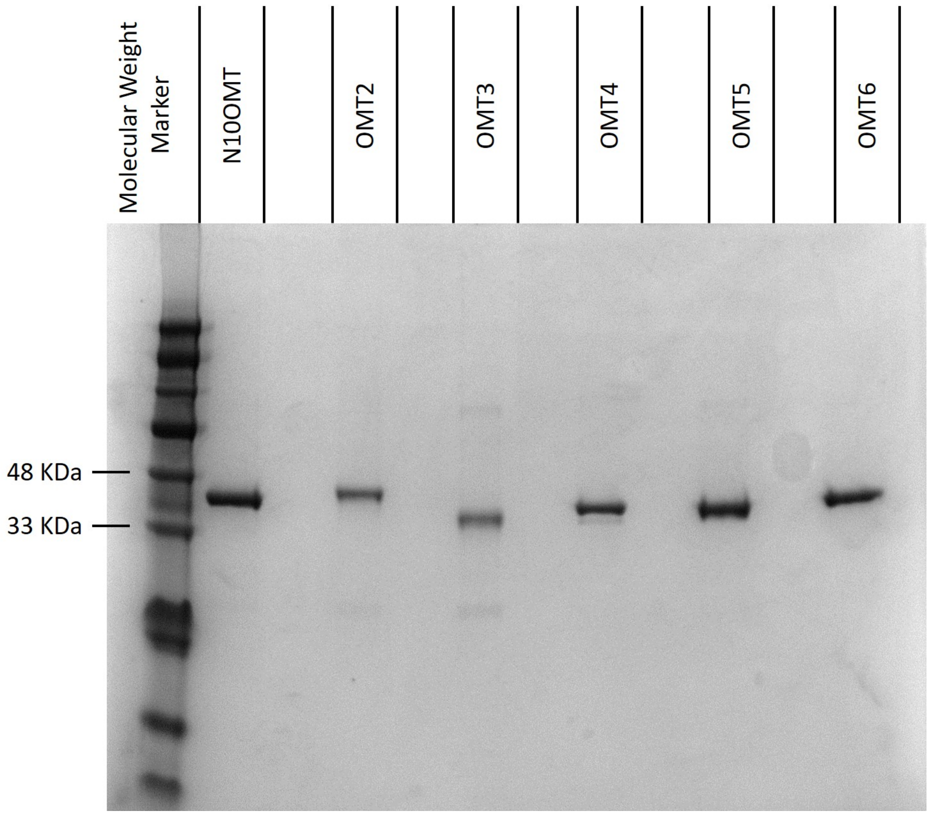


**Figure S4:** SDS-PAGE of recombinant OMTs produced in *Escherichia coli*. The left lane contains molecular weight protein markers and corresponding sizes are indicated to the left of the panel. All other lanes feature purified protein from *E. coli* strain soluBL21. Purification of polyhistidine-tagged recombinant proteins was achieved using a Nickel-affinity column and size exclusion chromatography. Visualization was achieved using Coomassie blue staining.
